# Genetic and epigenetic profiling of the infertile male

**DOI:** 10.1371/journal.pone.0214275

**Published:** 2019-03-21

**Authors:** Stephanie Cheung, Alessandra Parrella, Zev Rosenwaks, Gianpiero D. Palermo

**Affiliations:** The Ronald O. Perelman and Claudia Cohen Center for Reproductive Medicine, Weill Cornell Medicine, New York, New York, United States of America; Universite Clermont Auvergne, FRANCE

## Abstract

Evaluation of reproductive quality of spermatozoa by standard semen analysis is often inadequate to predict ART outcome. Men may be prone to meiotic error and have higher proportion of spermatozoa with fragmented chromatin, capable of affecting the conceptus' health. In men with unexplained infertility, supplementary tests may be pivotal to gain insight into the paternal contribution to the zygotic genome. A total of 113 consenting men were included in the study, with an additional 5 donor specimens used as control. Among study participants, 87 were screened for sperm aneuploidy by fluorescent in situ hybridization (FISH) and ranked according to their increasing age. A total of 18 men were assessed by whole exome sequencing and categorized according to their reproductive outcome as either fertile or infertile. Another set of men (n = 13) had their gene expression analyzed by RNA-seq and were profiled according to their reproductive capacity. FISH revealed that the average aneuploidy rate was highest for men over-55 age group (9.6%), while men >55 had the highest average disomy for chromosomes 17(1.2%) and 18(1.3%). ART results for the entire cohort comprised 157 cycles, stratified by paternal age. The youngest age group (25–30 years) had a fertilization rate of 87.7% which decreased to 46.0% in the >55 age group. Clinical pregnancy rate was highest in the 25-30yr group (80.0%) while no pregnancies were attained in the >55 age groups. Pregnancy loss was characterized by a steadily increasing trend, highest in the 51–55 age group (50.0%). NGS was performed on a cohort of patients classified as having recurrent pregnancy loss. This cohort was classified as the infertile group (n = 10) and was compared to a control group (n = 8) consisting of patients successfully treated by ART. Eight couples in 17 ICSI cycles achieved a clinical pregnancy rate of 82.4% while 10 infertile couples treated in 21 cycles achieved a pregnancy rate of 23.8%, all resulting in pregnancy loss. DNA-sequencing on spermatozoa from these patients yielded overall aneuploidy of 4.0% for fertile and 8.6% for the infertile group (*P*<0.00001). In the infertile cohort, we identified 17 genes with the highest mutation rate, engaged in key roles of gametogenesis, fertilization and embryo development. RNA-seq was performed on patients (n = 13) with normal semen analyses. Five men unable to attain a pregnancy after ART were categorized as the infertile group, while 8 men who successfully sustained a pregnancy were established as the fertile control. Analysis resulted in 86 differentially expressed genes (*P*<0.001). Of them, 24 genes were overexpressed and 62 were under-expressed in the infertile cohort. DNA repair genes (APLF, CYB5R4, ERCC4 and TNRFSF21) and apoptosis-modulating genes (MORC1, PIWIL1 and ZFAND6) were remarkably under-expressed (*P*<0.001). Sperm aneuploidy assessment supported by information on gene mutations may indicate subtle dysfunctions of the spermatozoon. Furthermore, by querying noncoding RNA we may gather knowledge on embryo developmental competence of spermatozoa, providing crucial information on the etiology of unexplained infertility of the infertile male.

## Introduction

Between 14 and 15% of couples of reproductive age present with infertility [1]. The American Society for Reproductive Medicine indicates that the contribution to the inability to reproduce is equally attributed to the male and the female partner, with the remainder being due to a combination of factors [2]. It is advisable that couples seek professional care after approximately 12 months of unprotected intercourse without achieving a pregnancy [3, 4].

In order to address the contribution of each partner to establish the appropriate clinical treatment and minimize the risk of failure, a primary evaluation of the female partner is often followed by an assessment on the man. If the workup for both partners is unrevealing, procedures such as timed intercourse (TIC) and intrauterine insemination (IUI) are proposed, with or without ovarian stimulation. Generally, after three or four unsuccessful IUI cycles and if even a mild abnormality in the workup of the female or male partner are noted, in vitro fertilization (IVF) with or without intracytoplasmic sperm injection (ICSI) is generally contemplated.

Regarding the evaluation of the male partner, it is current clinical practice to focus on the presence of an adequate number of spermatozoa in the ejaculated specimen with adequate motility and morphology capable of providing chances of fertilization. The usefulness of the semen analysis, however, in indicating the appropriate insemination treatment for couples with unexplained infertility appears limited [3, 5]. Moreover, a semen analysis rarely predicts the functioning or fertilizing capacity of the male gamete. This is particularly evident in cases with unexplained infertility where both the male and female partners have normal results for all conventional tests. Due to these reasons, several investigators have begun to explore the genetic causes of male infertility and the utilization of additional tests to gain more insight towards the reproductive capacity of the individual. For instance, ancillary tests such as the DNA fragmentation assay may be useful [6].

The difficulty in evaluating male gamete competence may be due to the fact that spermatogenesis is a complex differentiation process commonly divided into three main phases: self-renewal and proliferation of spermatogonia, meiotic division of spermatocytes, and post-meiotic differentiation of spermatids into spermatozoa. These events are controlled by well-coordinated transcriptional and post-transcriptional regulators. Furthermore, the male gamete is not just a carrier to deliver the male genome to the oocyte. During fertilization, the spermatozoon provides a highly structured genome with specific epigenetic markers defined by RNA and proteins that play an important role in proper embryo development [7–9].

An array of different studies have investigated an effect of genetic markers such as single-nucleotide polymorphisms (SNPs) [10], copy number variants (CNVs) [11], protamine content [12], methylation characteristics [13], protein content [14], and small RNAs [15, 16] in infertile men. However, these new assays are not used routinely in reproductive clinics. [17–21].

We hypothesize that through genetic and epigenetic analysis, we will be able to identify mutations and differential expression of specific genes in men presenting with unexplained infertility compared to those successfully treated by ART and a fertile control, leading to a better insight into the etiology of male infertility.

## Materials and methods

### Inclusion criteria and study design

A total of 113 sperm samples from male partners (38.3±7yrs) of couples with a history of prior ART failure or recurrent pregnancy loss were evaluated (S1 Fig), with an additional 5 donor specimens used as control. All patients, with a normal BMI (18.5–24.9), had peripheral karyotype [22] and chromatin fragmentation assessments [23] carried out. Semen parameters were evaluated according to the most recent WHO criteria [3]. Testicular sperm was obtained from testicular biopsies in patients with non-obstructive azoospermia and hypogonadism. Ejaculated specimens (n = 87) were prepared for FISH assessment to assess overall aneuploidy according to increasing paternal age. An additional analysis by DNA (n = 18) and RNA (n = 13) sequencing was carried out on spermatozoa to assess the copy number variants, gene mutations, and gene expression levels. We compared men treated by ART but unable to conceive (infertile group) to men who successfully achieved a clinical pregnancy (fertile group). Sperm analyses were not performed on the same specimens used for ART, and ejaculated spermatozoa assessed were collected immediately prior to the ART procedure. This study was approved by the Institutional Review Board of the New York Presbyterian Hospital-Weill Cornell Medicine (IRB 1006011085), and all patients gave informed written consent to participate.

### Spermatozoa collection and preparation

Ejaculates provided by masturbation were centrifuged in 3:1 dilution with human tubal fluid medium buffered with HEPES (H-HTF; Irvine Scientific, Santa Ana, CA) at 600*g* for 10 minutes on a single layer density gradient (Enhance-S Plus Cell Isolation Media, 90%; Vitrolife, San Diego, CA) to remove the seminal fluid. Testicular spermatozoa were retrieved via testicular biopsy, as previously described [24]. Five μl of specimen were smeared on a glass slide and allowed to dry overnight for FISH and TUNEL processing. For DNA and RNA sequencing, a final suspension of the specimen at 1-2x10^6^/ml was prepared.

### Preparation of spermatozoa for FISH analysis

For FISH analysis, 5μl of washed semen or surgically retrieved spermatozoa was smeared on precleaned glass slides and allowed to dry overnight. Slides were fixed in Carnoy’s fixative (3:1 methanol: acetic acid) for 15 minutes at room temperature, and placed on a 37°C slide moat overnight. Sperm nuclei were decondensed by slide incubation for 3 minutes at room temperature in 5 mmol/L dithiothreitol (DTT; Sigma Chemical Co., St. Louis, MO) in 0.1M tris(hydroxymethyl)aminomethane (Trizma HCl; Sigma Chemical Co.), followed by 3M Sodium Chloride and 300mM tri-Sodium citrate dehydrate (2X standard saline citrate; Vysis, Downers Grove, IL) pH 7.0 for 1 minute at room temperature. Excessive agitation of the slides was avoided in all decondensation and washing steps in an effort to limit sperm loss, especially from smears performed with testicular spermatozoa. Decondensed slides were hybridized with probes specific to chromosomes X, Y, 13, 15, 16, 17, 18, 21, and 22 for 5 minutes at 37°C. Sperm nuclei were then counterstained with 7 ul of 4’,6-diamino-2-phenylindole (DAPI) and cover-slipped. Using an Olympus BX61 fluorescent microscope at 1000X, incidences of disomy, nullisomy, and diploidy was assessed in each specimen for at least 1000 spermatozoa to increase accuracy, with a 1.6% threshold. The same assessment was done on anonymous donor controls. Slides were also processed and assessed in replicate to maintain a 2–3% FISH error (Applied Imaging, CytoVysion v3.93.2).

### Sperm chromatin assessment

The terminal deoxynucleotidyl transferase dUTP nick end labeling (TUNEL) was carried out according to a protocol previously reported [25]. Briefly, 5μl of raw semen sample was smeared on precleaned glass slides and allowed to dry overnight. Slides were then fixed in 4% paraformaldehyde for one hour and left to dry once more overnight. The slides were permeabilized in a solution of 0.1% Triton X-100 and 0.1% Sodium Citrate in PBS for two minutes, washed, and allowed to dry. The kit reagent was then added to the slides (In Situ Cell Death detection Kit; Roche Diagnostics, Rotkreuz, Switzerland), a coverslip added, stored in a humidified chamber at 37°C for one hour and subsequently washed with PBS. Upon drying, 7μl of DAPI antifade solution was added to counterstain nuclei and coverslips applied. Slides were screened under a fluorescent microscope for green fluorescence indicating chromatin fragmentation, with a threshold of 15%.

### Whole molecular karyotype by NGS

Sperm specimens were processed by centrifugation in human tubal fluid (HTF; Irvine Scientific, Santa Ana, CA) at 600*g* for 10 minutes. After adjusting the concentration to 500 cells/mL for each sample, DNA extraction and amplification was achieved with the use of a commercial kit (Repli-G Single Cell; Qiagen, Hilden, Germany) through PCR-based random hexamer amplification. Decondensation was carried out by incubating specimens with Dithiothreitol (DTT) at 65°C for 10 minutes. Following amplification, DNA was submitted for quality control testing where a DNA concentration of 447.8±198ng/ul and purity of 1.7±0.1 was confirmed. DNA specimens were then sent to an external facility (Genewiz, Inc; South Plainfield, NJ), where they were processed by 150-bp-paired-end sequencing on an Illumina HiSeq 2500 system, 2 samples per lane. After the sequenced reads were trimmed to remove nucleotides with poor quality (error rate <0.01), they were aligned to the human reference genome (hg20) with CLC Genomics Server 9.0. Quality assessments of each indexed sample were performed using QPCR, and high quality coverage of 85x was obtained for each specimen (Agilent SureSelect Human All Exon V6) with at least 90% exome coverage. Base calling accuracy for all samples was ~99.9%, as indicated by an average Phred score of Q38. Following CNV detection, completed using CLC Genomics Server 9.0, detected variants were compared with the single nucleotide polymorphism database to filter out common variants. Remaining variants were then further annotated and used for the detection of gene mutations. Genes were considered duplicated when the read depth was greater than 1.5 times the median depth in the control group for more than 70% exons in the gene, and were considered deleted when the read depth was less than 0.5 times the median depth in the control.

### RNA extraction in human spermatozoa

Seven to 25x10^6^ human sperm cells were lysed by β-mercaptoethanol for 5 minutes, and total RNA was isolated using a hybrid protocol with warmed (37°C) TRIzol Reagent (ThermoFisher Scientific, USA) and homogenized by vigorous vortexing. Ethanol (70%) was added to each tube of the homogenized cells and mixed well by pipette. Impurities were removed with RW1 buffer, followed by RPE buffer (Qiagen, Hilden, Germany). Total RNA was purified using an RNeasy Mini Kit spin column (RNEasy; Qiagen, Hilden, Germany), at room temperature. Ribonucleic acid was eluted with 30μl of RNase free water directly onto the columns followed by a spin at 11,000 rpm at 4°C for 1 minute. The nucleic acid was quantified by an Agilent 2100 bioanalyzer to determine RNA integrity number (RIN). Spermatozoal RNA concentration was calculated by a NanoDrop spectrophotometer and confirmed using Qubit RNA assay. Library prep was carried out (NEBNext Ultra RNA Library Prep kit, New England BioLabs Inc., Ipswich, MA) with an insert size of ~188–226 bp (S2 Fig), and ribosomal RNAs were isolated from total RNA using rRNA depletion (Ribo-Zero Gold rRNA Removal kit, Illumina, San Diego, CA). To confirm the quality of potential reads, a pilot paired-end 36bp RNA-sequencing was carried out (NextSeq 500; Illumina, San Diego, CA) by an external laboratory (Genewiz, Inc; South Plainfield, NJ) and expanded to 50–60 M reads at 2x75 bp. Sequenced reads were trimmed to remove low quality bases at ends and were then mapped to the reference genome (hg20) using CLC Genomics Server 9.0. For differential expression analysis, raw read counts were loaded as input according to the DESeq2 v1.23.1 (LGPL, Bioconductor) pipeline. Data quality control was then carried out to determine the total number of reads and the proportion of null counts for each sample in order to detect possible outliers. Then, data normalization was achieved, followed by gene expression comparison [26]. To avoid the possibility of over- or under-representing FPKM, an algorithm by edgeR (LGPL; Bioconductor) and CONTRA was implemented following the expression analysis by DESeq2, to overcome experimental conditions such as fragmentation.

### Ovarian stimulation and oocyte preparation

Ovarian superovulation was performed with gonadotropins and with GnRH-agonists or antagonists [27] [28]. The adoption of a particular superovulation protocol was chosen based on patient characteristics and prior history. Ovulation was triggered by human chorionic gonadotropin (hCG) when follicles had reached an adequate diameter, and oocyte retrieval was performed approximately 35 to 36 hours post hCG. ICSI was carried out in the standard fashion[28].

### Pregnancy assessment and luteal support

Beginning on the day of oocyte harvest, luteal support was carried out by methylprednisolone (16 mg/day) and tetracycline (250 mg every 6 hours) were administered for 4 days to all patients. Progesterone supplementation (25–50 mg I.M./day) began after retrieval and continued until the clinical pregnancy was established. Ten to 14 days after egg retrieval, a serum βhCG test was performed. A pregnancy was defined as biochemical when the βhCG level decreased prior to an implantation site visible at ultrasound. Clinical pregnancy was entailed by visualization of a fetal heartbeat (+FHB) by ultrasound assessment during the 7th week of gestation.

### Data analysis

The CNV assessment was done using CLC Genomics Server 9.0 modules including NGS Core Tools/Mapping and Re-sequence analysis. Coverage data obtained from CLC Genomics was used for the CNV analysis by programs developed within Genewiz. Study group copy number gains and losses were compared to base-level log-ratios created from the control group. The CNVs were then ranked according to these log-ratio values and the corresponding genes were noted and grouped by function. RNA analysis with specific focus on non-coding RNA was performed using the open-source software bioinformatics tool DESeq2 v1.23.1 (LGPL, Bioconductor) to measure the abundance of long non-coding RNA (lncRNA) that were previously known and annotated from the human genome (hg20). Differential gene expression analysis was carried out by DESeq2, and gene expression values were calculated in Fragments Per Kilobase of Exon of transcript per million mapped reads (FPKM). Statistical thresholds of *P*<0.0005 for significance and *Q*<0.05 threshold for false positive discovery were used. Friedman’s Chi-square analysis with the Yate’s correction using the Sigma Stat program (Jandel Scientific, San Rafael, CA) was used to evaluate the aneuploidy rates, as well as the CNVs and gene expression values between the study and control groups. Pearson’s correlation coefficient and unpaired *t*-test were also used to compare the control and study groups (Graphpad Software, San Diego, CA). A *P* value of <0.05 was considered to be statistically significant.

## Results

**Fig 1:**
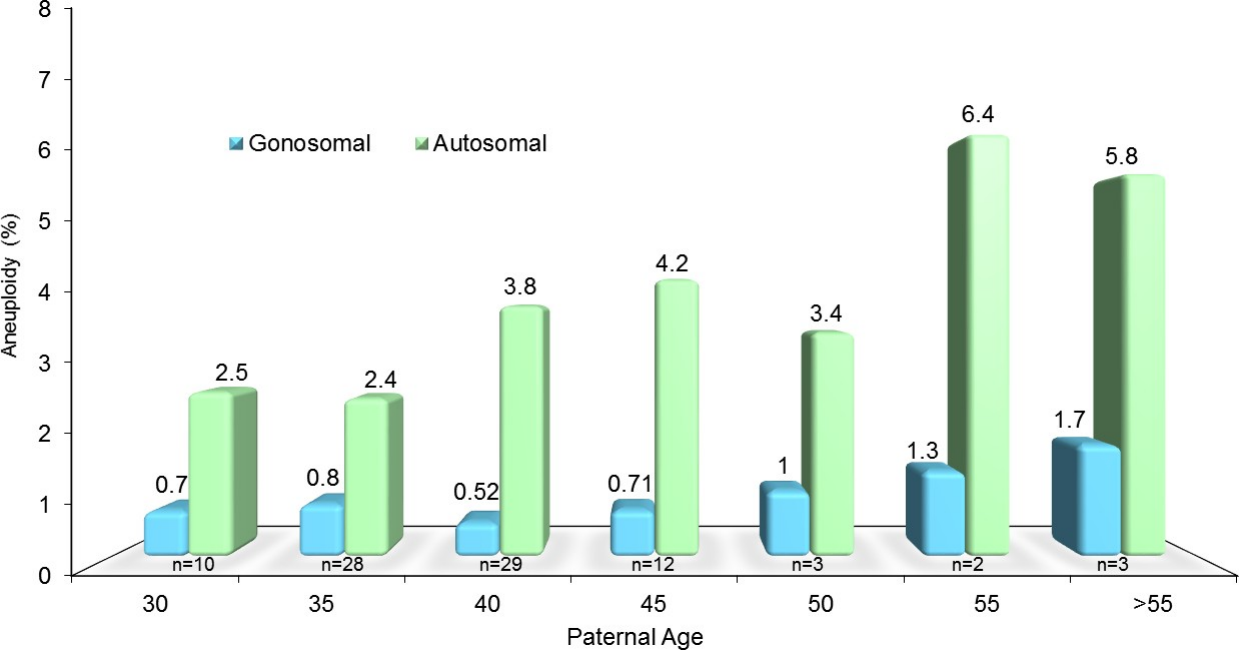
Sperm aneuploidy assessment by FISH depicting sperm chromosomal defects. A comparison between autosomal aneuploidy and male age, assesed by Pearson's Correlation Coefficient, had an R^2^ value of 0.6 (*P*<0.00001). Data has been allocated into different age bins, as shown, for clinical relevance and visual representation.

**Fig 2:**
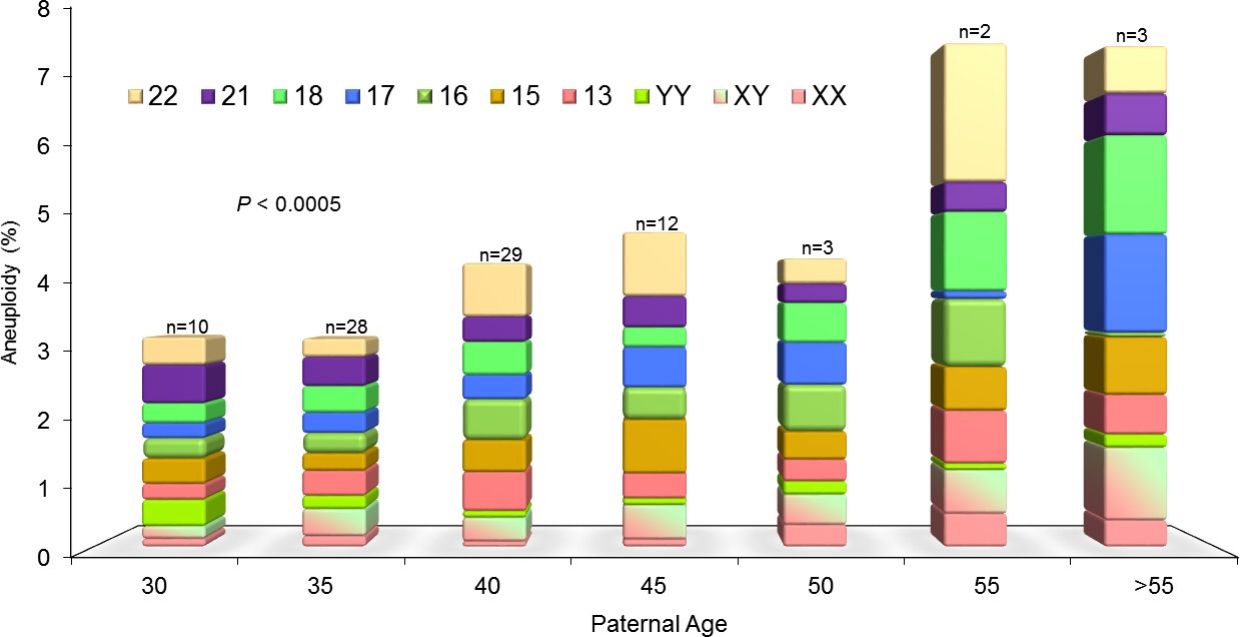
FISH aneuploidy assessment by individual chromosomes in relation to advancing male age. Of particular note were chromosomes 15 (orange) and 17 (blue), which progressively increased with age, while chromosome 21 (purple) was noticeably highest in the youngest age group, progressively decreasing with advancing male age.

In men who have apparently normal semen parameters, the inability to support a successful pregnancy indicates a concealed abnormality that may impair gamete competence. In these 113 men with a normal peripheral karyotype and a chromatin fragmentation of 18.2 ± 8% (range 8.6–32.4%), the quest for the origin of male infertility can be initiated with the assessment of the gamete chromosomal status. Interestingly, there was a progressive increase in aneuploidy of the gonosomes but most importantly of the autosomes in relation to advancing male age (Fig 1). While this appears more dramatic for specific chromosomes such as Chr 15 and 17, we noticed that Chr 21 abnormalities appeared higher in younger men (Fig 2).

**Fig 3:**
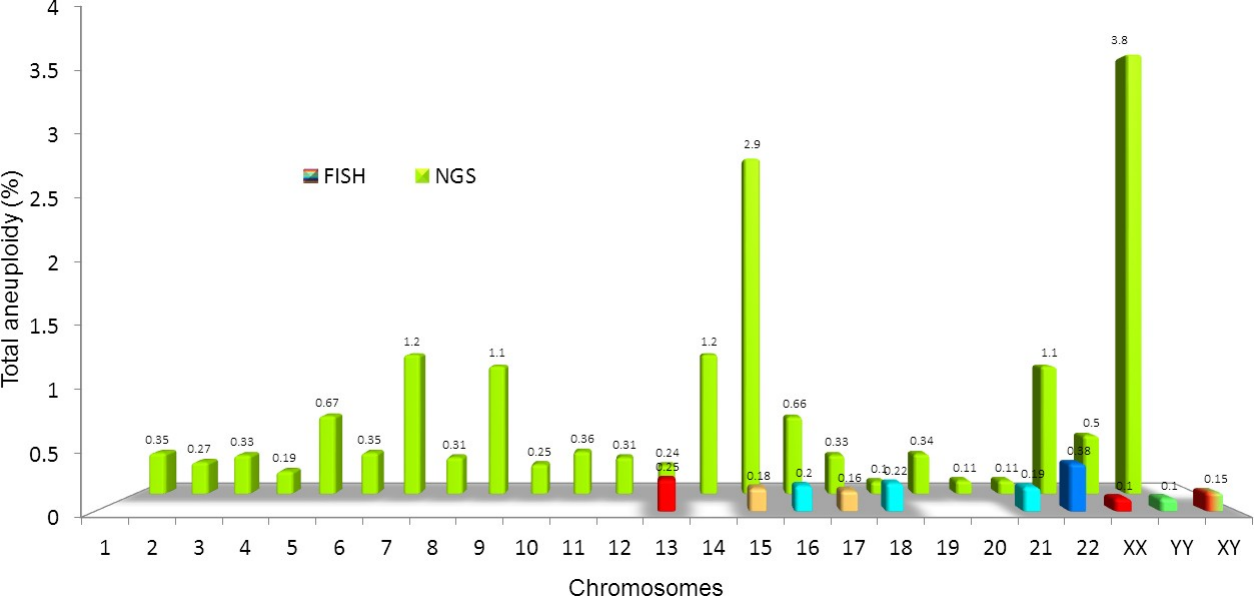
Assessment of sperm aneuploidy for each chromosome by two different methods. In the first row, the minute multicolor columns indicate the chromosomal defects assessed by FISH on 9 chromosomes. In contrast, the green histogram represents whole genome molecular karyotyping by NGS. This technique allows for a more accurate and comprehensive assessment of all chromosomes to detect sperm aneuploidy.

These findings were supplemented by assessing specific copy number variants of sequenced DNA in individual spermatozoa. Indeed, with this approach all chromosomes were sequenced, resulting in a more accurate aneuploidy assessment (Fig 3). Semen specimens of men unable to conceive (n = 10) were compared to fertile individuals (n = 8). The group defined as infertile retained the ability to achieve fertilization and support the development of conceptuses capable of implanting, but resulting in pregnancy loss (Table 1).

**Table 1:** Couples’ demographic, gamete characteristics and ICSI outcome.

	Control	Infertile
**Couples**	8	10
**Male Age (M yrs ± SD)**	34.6 ± 2	37.9 ± 5
***Semen Parameters***		
Concentration (10^6^/mL ± SD)	25.6 ± 31	22.9 ± 28
Motility (% ± SD)	29.2 ± 29	23.7 ± 31
Morphology (% ± SD)	1.5 ± 2	1.4 ± 2
**Cycles**	17	21
**Clinical Pregnancy (+FHB)**	14 (82.4)*	5 (23.8)*
**Pregnancy Loss (%)**	-	5 (100)

* χ^2^, 2x2, 1 *df*, *P*<0.05

+FHB: Presence of 1 fetal heartbeat

**Fig 4:**
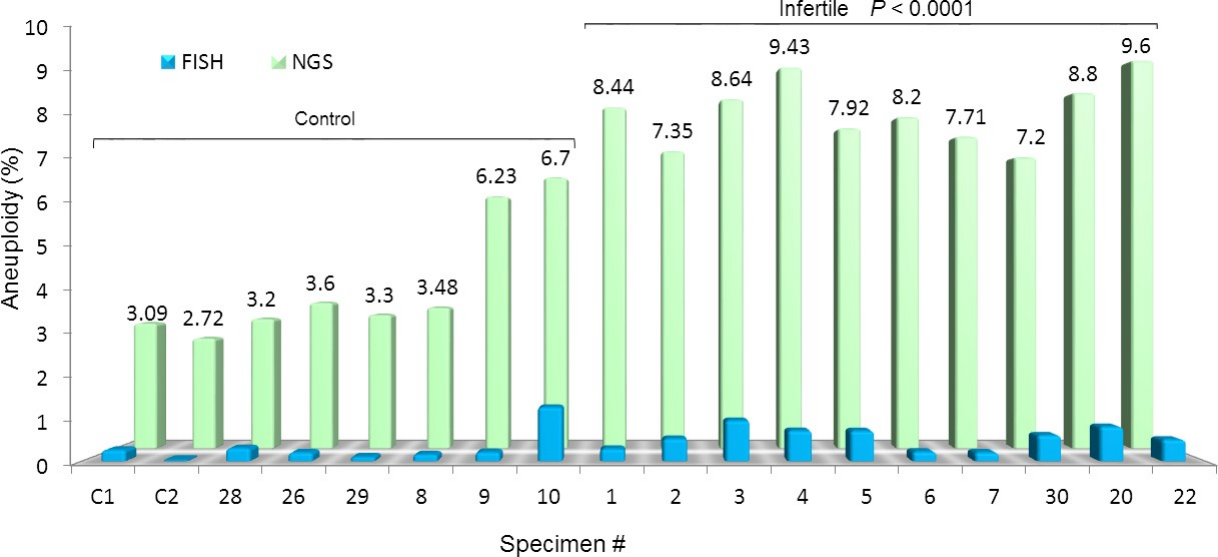
Aneuploidy assessment by FISH and NGS in the study population. FISH analysis, depicted by the blue histogram, failed to evidence any difference among the groups. NGS, however, reported a remarkable and significant aneuploidy rate in the infertile group when compared to the fertile control (*P*<0.0001).

**Fig 5:**
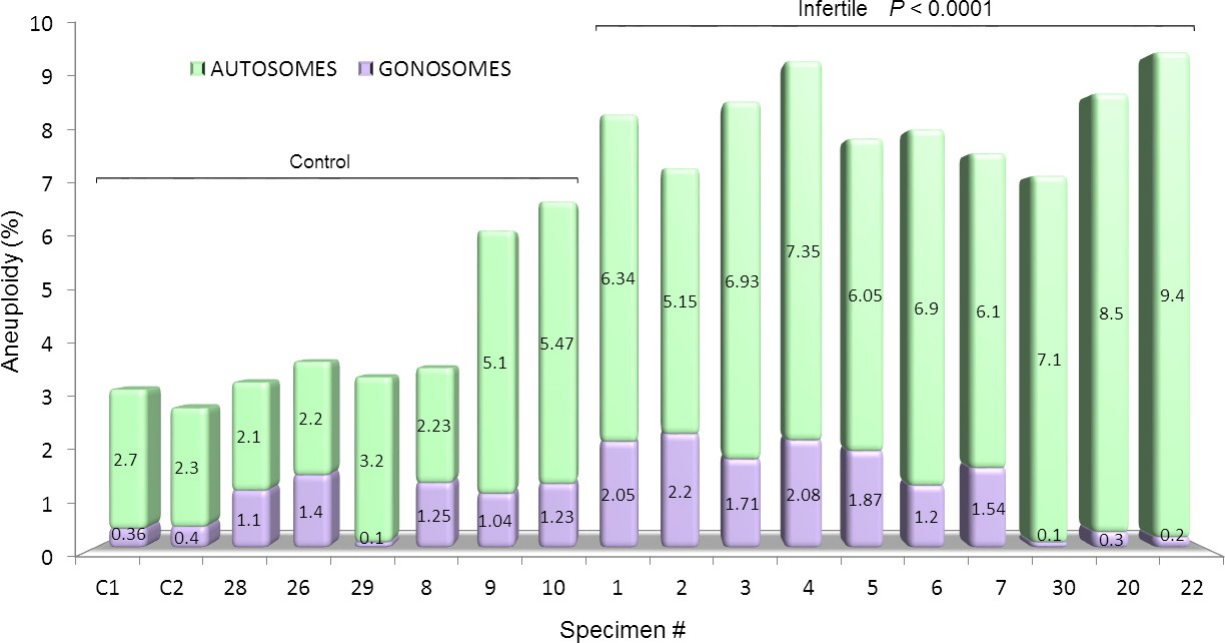
NGS assessment of sperm aneuploidy according to chromosome type in the study population. Both autosomal and gonosomal chromosomes contributed to the overall higher aneuploidy of the infertile cohort in comparison to the fertile control group (*P*<0.0001). The most represented chromosomal defects were related to the autosomes (green portion of the histogram).

While the standard FISH technique was unable to disclose differences among the groups of men studied (Fig 4), DNA sequencing evidenced that overall aneuploidy was increased in men successfully treated by ART and even higher in infertile men (*P*<0.0001) when compared to the control. Furthermore, a closer assessment also indicated a significantly higher gonosomal and autosomal aneuploidy in the infertile cohort (*P*<0.0001) when compared to the control (Fig 5). An advantage of sequencing technology is the ability to identify specific genes and whether there are mutations involved. According to the highest CNV observed, we depicted 11 genes. Their action ranged from general ancillary cellular function in the testis to meiotic crossing-over regulation (*RBMY1F*), sperm development (*DPY19L2*), involvement in sperm-egg fusion (*ADAM3A*), and nucleus-cytoplasmic transport of RNA (*NXF2*) (Table 2).

**Table 2:** List of genes with the largest CNV imbalance according to chromosomes and listed by function.

Gene	Chr	Function
*DPY19L2*	7	Sperm head elongation and acrosome formation
*ADAM3A*	9	Involved in sperm-egg fusion during fertilization
*HAUS1*	18	Mitotic spindle assembly, maintenance of centrosome integrity, completion of cytokinesis
*KIF4A*	X	Mitotic chromosomal positioning; bipolar spindle stabilization
*XRN1*	3	Homologous recombination, meiosis, telomere maintenance, and microtubule assembly
*NLRP7*	19	Trophoblast development
*SIRPB1*	20	Recruitment of tyrosine kinase SYK
*NXF2*	X	mRNA export from the nucleus to the cytoplasm
*TEX11*	X	Regulator of crossing-over during meiosis
*CSF2RA*	Y	Activation of hematopoietic cells
*RBMY1F*	Y	Sperm development, pre-mRNA splicing in the testis

**Fig 6:**
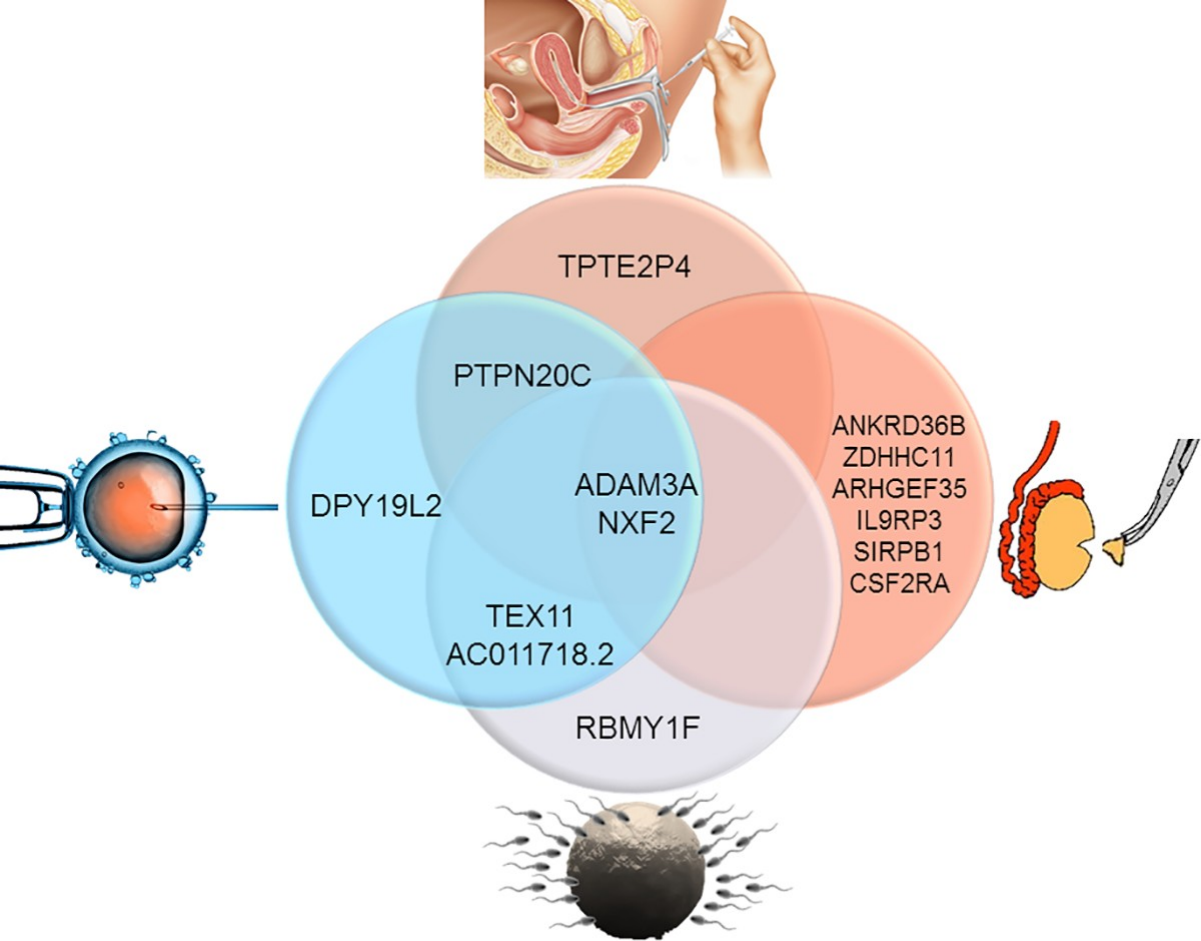
Gene mutation assessment according to ART treatment. Several gene mutation commonalities were identified when taking into consideration the type of assisted reproduction technique used. In couples treated by IUI, only a pseudogene (*TPTE2P4*) was identifed while a gene involved in spermatogenesis (*RBMY1F*) were mutated in those treated by IVF. A gene supporting sperm acrosome formation (*DPY19L2*) was mutated in men requiring ICSI. When we assessed gene mutations in men treated by testicular biopsy, a variety of genes were identified that play a role in basic cellular functions (*ANKRD36B*), androgen production modulation (*SIRPB1*), or activation of germ cells (*CSF2RA*). Common gene mutations were also identified in patients who underwent multiple types of ART treatments. For instance, men treated by both IVF and ICSI shared an imbalance of *TEX11*, a gene involved in meiotic crossing-over. All couples possessed gene mutations in *ADAM3A* and *NXF2*, which support sperm-egg fusion and RNA nucleus-cytoplasmic transport, respectively.

When considering the CNV gene imbalances of couples treated by different assisted reproduction techniques, we identified several gene mutation commonalities (Fig 6). Indeed, in couples who were treated by IUI (n = 6), only pseudogenes were involved (*TPTE2P4*), while for the couples treated by in vitro insemination (IVF) (n = 5) a gene involved in spermatogenesis was imbalanced (*RBMY1F*). In men requiring ICSI (n = 16), a gene (*DPY19L2*) involved in supporting specific functional components of the male gamete such as the acrosome was mutated. For men treated by testicular biopsy, a larger number of genes were involved in contributing to basic cellular functions (*ANKRD36B*), androgen production modulation (*SIRPB1*), or activation of germ stem cells (*CSF2RA*). Interestingly, men treated by IVF and ICSI shared in common imbalance of a gene involved in meiotic crossing-over. Finally, all couples requiring some forms of assisted conception had mutations in *ADAM3A* and *NXF2*, genes responsible for supporting sperm-egg fusion and RNA nucleus-cytoplasmic transport, respectively.

**Fig 7:**
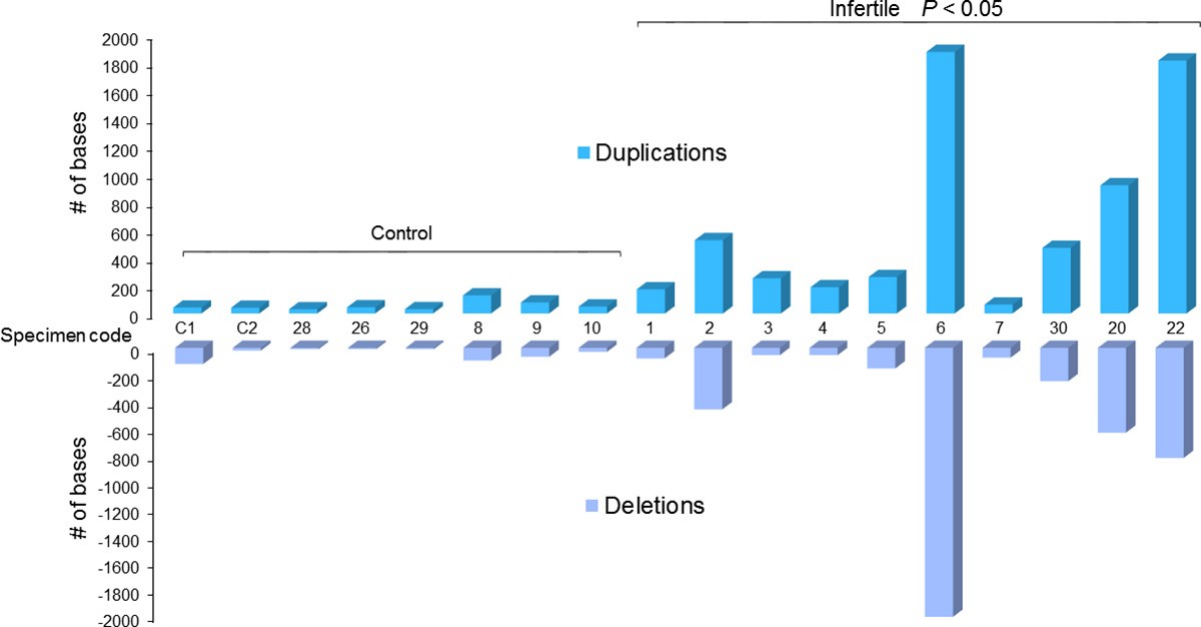
Histogram graph displaying overall gene mutations observed by CNV analysis in the study population. Each bar indicates a specimen. The gene duplications (dark blue histogram) and gene deletions (light blue histogram) are higher in the infertile cohort in comparison to the fertile control group (*P*<0.05).

An analysis of gene duplications and deletions confirmed a progressive increase in genomic mutations (*P*<0.05) for men with a reduced or compromised ability to generate offspring (Fig 7) when compared to the fertile individuals and control.

**Fig 8:**
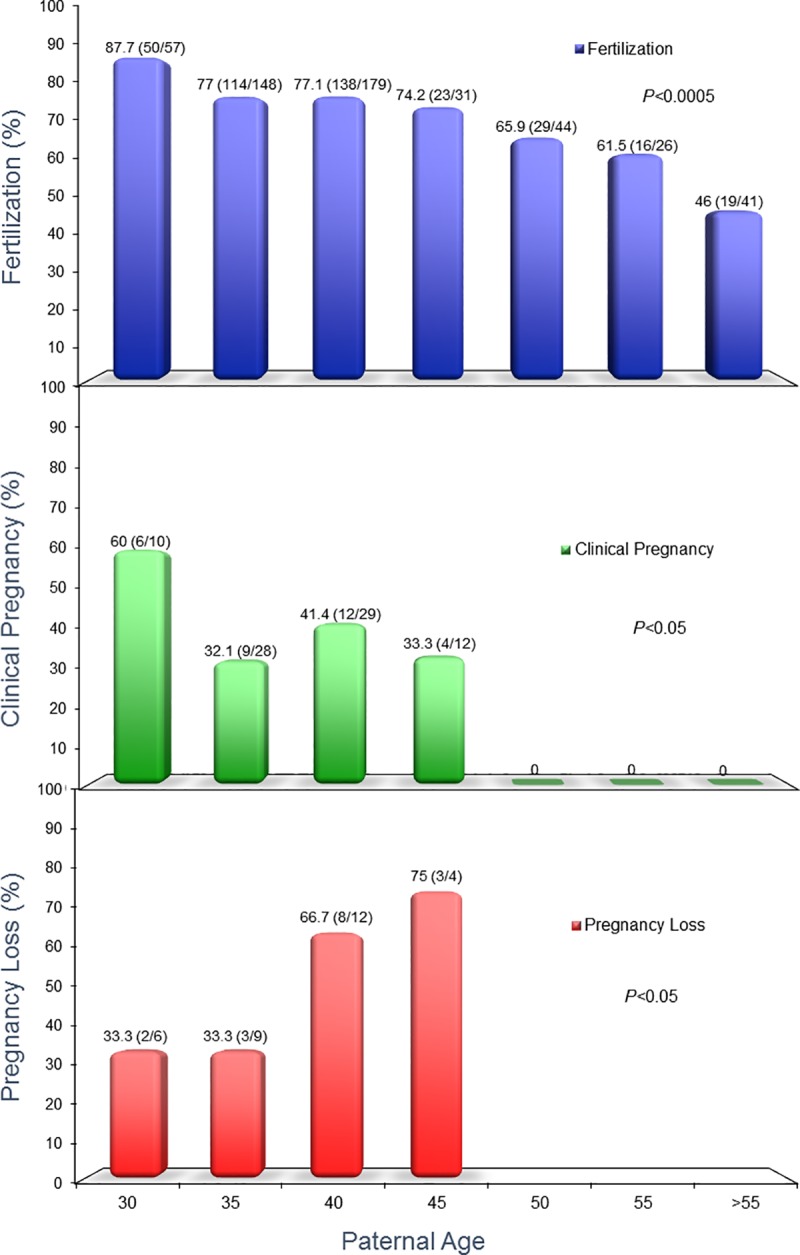
Fertilization, clinical pregnancy, and rate of pregnancy loss in relation to paternal age. ICSI outcome was assessed for men whom we performed a FISH assessment on. Fertilization was characterized by a decreasing trend and was lowest in the >55 age group. Clinical pregnancy rates was highest in the 25–30 age group but became absent in the 51–55 and >55 age groups. The rate of pregnancy loss was also characterized by an increasing trend with advancing paternal age.

Age can also affect fertility, even if it is not clear whether the culprit is cell aging itself or the consequence of an environmental insult that has manifested with the advancing aging process of the individual. Indeed, when we assessed the ICSI outcome of those men for whom we performed a FISH aneuploidy assessment, controlling for female age, we found that the fertilization rate was characterized by a decreasing trend, ranging from 87.8–46%, and was lowest in the >55 age group. Similarly, clinical pregnancy rate was highest in the 25–30 age group (81.0%) but was absent in the 50–55 and >55 age groups. Furthermore, the rate of pregnancy loss was characterized by a steadily increasing trend, highest in the 51–55 age group (50.0%) (Fig 8). These epigenetic insults vary, particularly regarding the male gamete, from the ability of the spermatozoon to carry a cytosolic factor capable of activating the oocyte to the ability to provide a functional centrosome that will ordain the chromosomal segregation at the first mitotic division of the conceptus. In this study, we queried the expression of specific genes involved in meiotic spermatogenesis and those involved in supporting the formation of the sperm cell in its fully functional form by assessing the gene expression of sperm specimens from 13 individuals. Among them, we specifically compared men treated by ART but unable to conceive to men who successfully achieved a pregnancy through assisted reproduction (Table 3).

**Table 3:** Couples’ demographic, gamete characteristics and pregnancy outcome.

	Control	Infertile
**Couples**	8	5
**Male Age (M yrs ± SD)**	32.8 ± 4	37.6 ± 3
***Semen Parameters***		
Concentration (10^6^/mL ± SD)	42.4 ± 13	45.3 ± 15
Motility (% ± SD)	44.2 ± 11	45.2 ± 14
Morphology (% ± SD)	2.9 ± 2	2.7 ± 2
**Cycles**	8	5
**Fertilization (%)**	23/29 (79.3)	30/42 (71.4)
**Clinical Pregnancy (+FHB)**	8	0
**Delivered**	8	0

**Fig 9:**
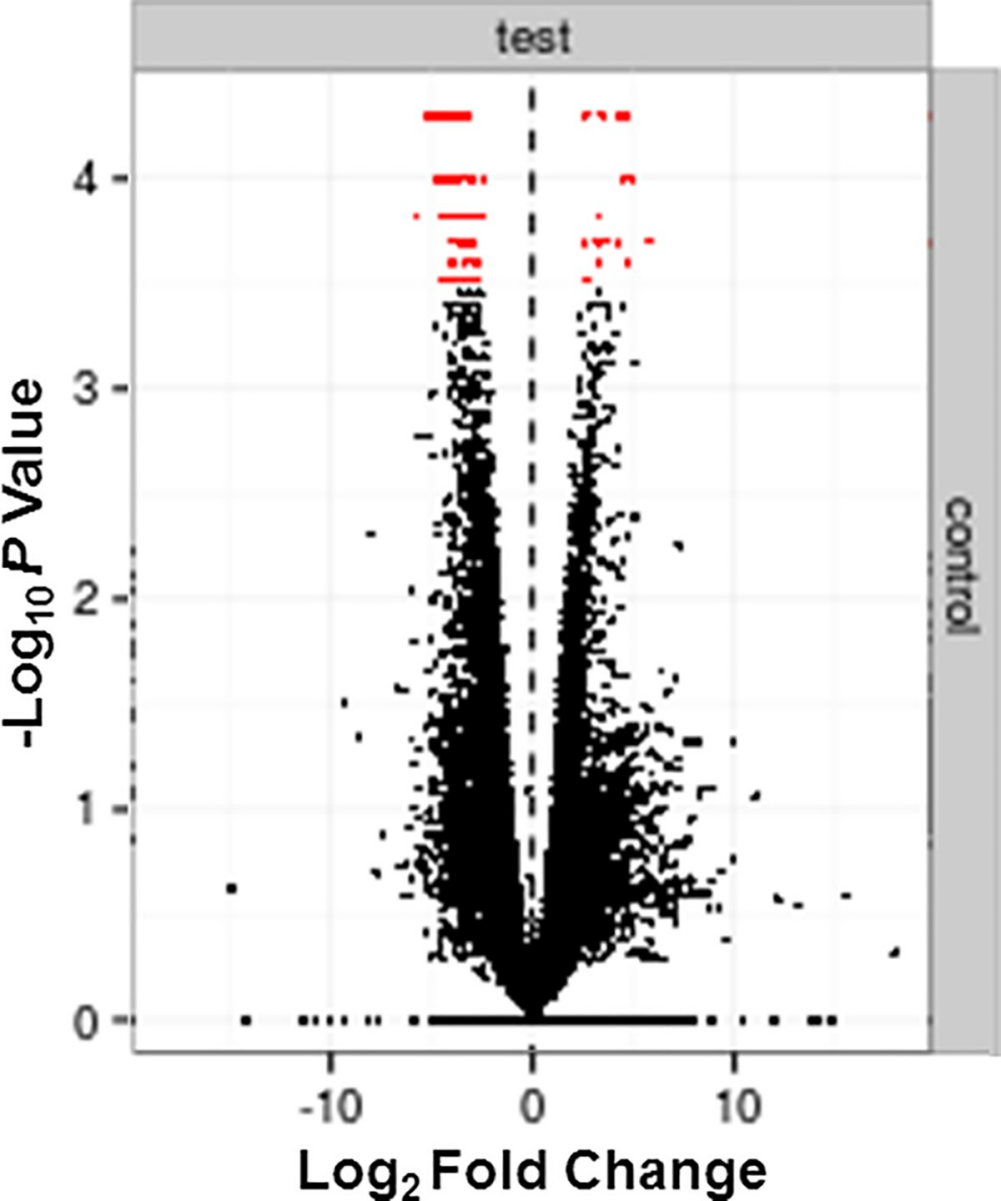
Volcano plot indicating the significantly imbalanced (in red) genes in logarithmic fold of RPKM. We identified a total of 24 over-expressed genes on the right of the dotted line, and 62 under-expressed genes on the left.

**Fig 10:**
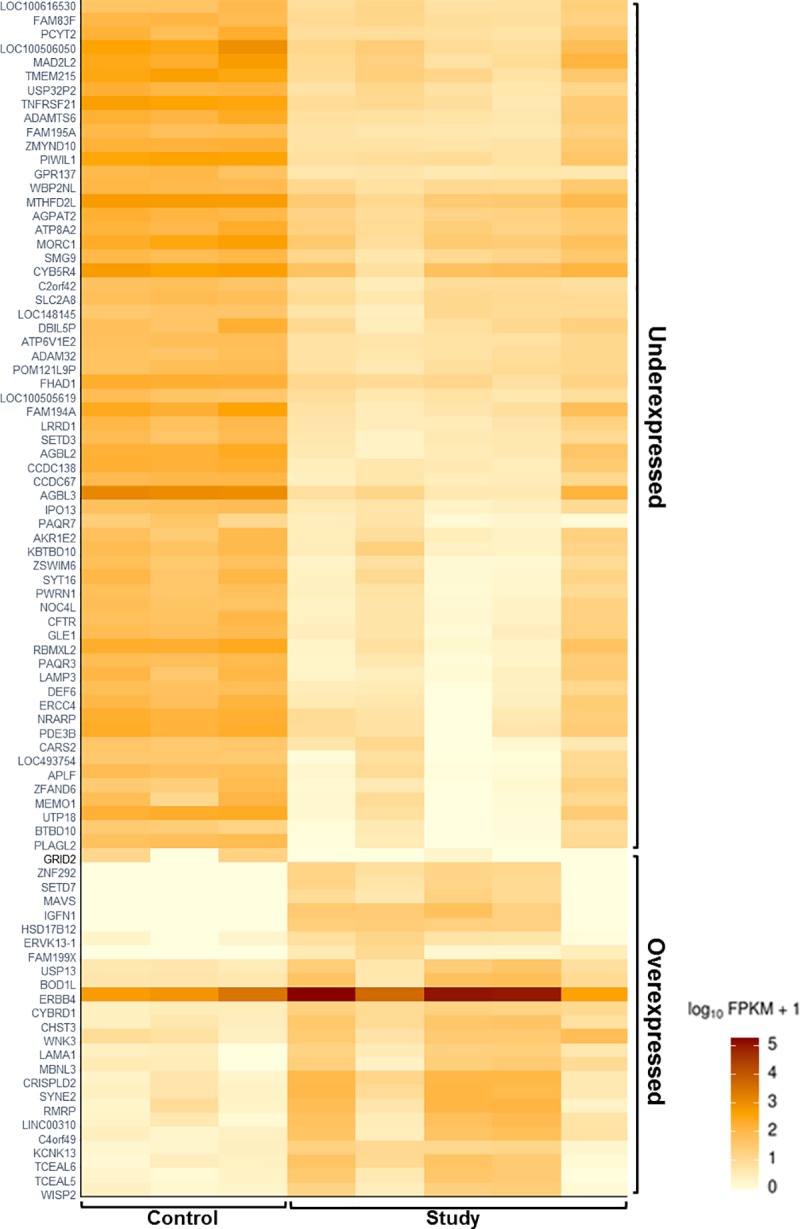
Heat map depicting the differentially expressed genes (by RNA analysis in spermatozoa) in the study group versus the reference control. Within the study group, there were 24 over-expressed (bottom right) and 62 under-expressed genes (top right) identified.

Genes with a *P*-value threshold of <0.05 were called as differentially expressed. Following paired-end 75 bp sequencing of the extracted spermatozoa RNA to assess about 23,260 genes, we identified 86 of them as imbalanced compared to donor controls (Fig 9), 24 of which were actually overexpressed while predominantly 62 were indeed underexpressed (Table 4, Fig 10, S1 Table).

**Table 4:** Differentially expressed genes.

Underexpressed Genes			Overexpressed Genes
*LOC100616530*	*ATP6V1E2*	*SMG9*	*ZNF292*
*FAM83F*	*ADAM32*	*CYB5R4*	*SETD7*
*PCYT2*	*POM121L9P*	*C2orf42*	*MAVS*
*LOC100506050*	*FHAD1*	*SLC2A8*	*IGFN1*
*MAD2L2*	*LOC100505619*	*LOC148145*	*HSD17B12*
*TMEM215*	*FAM194A*	*DBIL5P*	*ERVK13-1*
*USP32P2*	*LRRD1*	*DEF6*	*FAM199X*
*TNFRSF21*	*SETD3*	*ERCC4*	*USP13*
*ADAMTS6*	*AGBL2*	*NRARP*	*BOD1L*
*FAM195A*	*CCDC138*	*PDE3B*	*ERBB4*
*ZMYND10*	*CCDC67*	*CARS2*	*CYBRD1*
*PIWIL1*	*AGBL3*	*LOC493754*	*CHST3*
*GPR137*	*IPO13*	*APLF*	*WNK3*
*WBP2NL*	*PAQR7*	*PWRN1*	*LAMA1*
*MTHFD2L*	*AKR1E2*	*NOC4L*	*MBNL3*
*AGPAT2*	*KBTBD10*	*CFTR*	*CRISPLD2*
*ATP8A2*	*ZSWIM6*	*GLE1*	*SYNE2*
*MORC1*	*SYT16*	*RBMXL2*	*RMRP*
*LAMP3*	*BTBD10*	*PAQR3*	*LINC00310*
*ZFAND6*	*PLAGL2*	*UTP18*	*C4orf49*
*MEMO1*	*GRID2*		*KCNK13*
			*TCEAL6*
			*TCEAL5*
			*WISP2*

**Fig 11:**
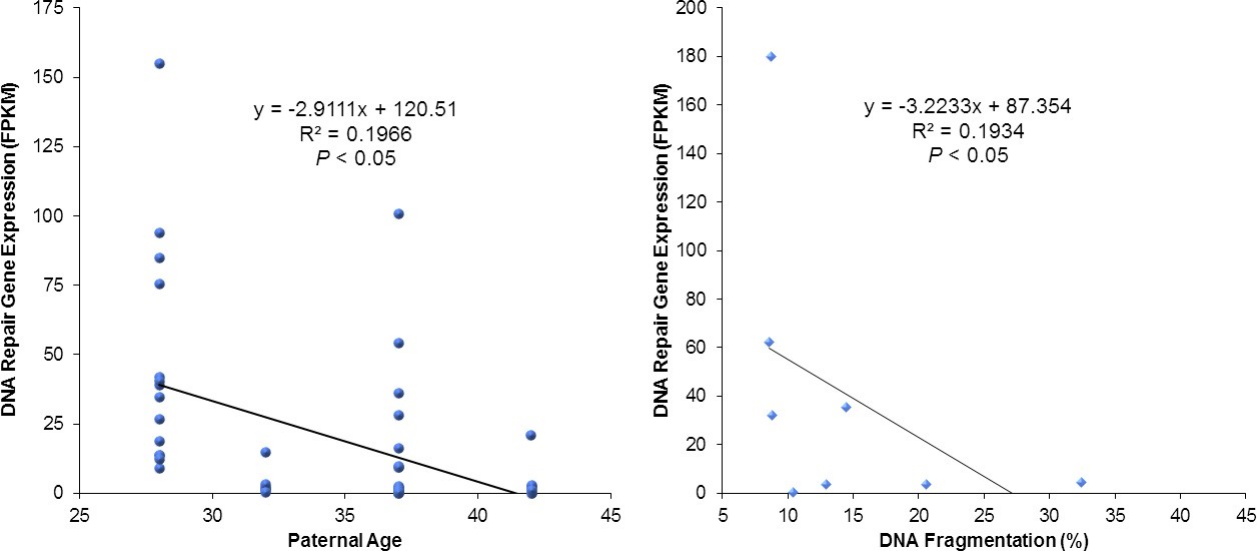
Effect of paternal age and DNA fragmentation on DNA repair gene expression. Assessment of the 7 most significant differentially expressed genes according to paternal age (left graph) showed an inverse correlation, with the oldest men having much lower expression of these DNA repair and apoptotic regulating genes (*P*<0.05). Similarly, plotting the expression of these genes in function of increasing sperm DNA fragmentation (right graph) illustrated that the higher the expression of genes related to DNA repair and apoptosis, the lower the sperm DNA fragmentation (*P*<0.05).

The overall function of these underexpressed genes involved nuclear compaction, centrosomal development, mitochondrial activity, as well as flagella development and function. A closer assessment identified 7 genes that were significantly underexpressed (*P*<0.001) (Table 5) and were mainly involved in apoptosis (*MORC1*, *TNFRSF21*, *ZFAND6*) and DNA repair mechanisms (*APLF*, *ERCC4*). These 7 genes also appeared to have an inverse correlation with male age as well as sperm DNA fragmentation (*r*_*s*_ = -0.5, *P*<0.05) (Fig 11).

**Table 5:** Significantly underexpressed genes.

Gene	Chr	Description	Log2 Fold Change	*P*
*APLF*	2	Single-strand and double-strand DNA break repair	-2.92	0.0002
*MORC1*	3	Affects entry into apoptosis	-4.08	0.0003
*CYB5R4*	6	Protects the cell from reactive oxygen species (ROS) buildup	-4.07	0.0001
*TNFRSF21*	6	Promotes apoptosis mediated by BAX	-4.52	0.0002
*PIWIL1*	12	Represses transposable elements	-4.44	0.0003
*ZFAND6*	15	Regulates TNF-alpha induced NF-kappa-B activation and apoptosis	-2.91	0.0003
*ERCC4*	16	Regulates 5' incision made during nucleotide excision repair	-3.16	0.0001

Among all genes assessed, we evidenced 28 (0.1%) ncRNAs that were differentially expressed (*P*<0.0005) between the control cohort (n = 8) and the infertile cohort, men (n = 5) treated by ICSI achieving a fertilization of 71.4% (30/42) but not capable of sustaining a pregnancy (Table 6). All 28 differentially expressed RNAs were classified as long intergenic non-coding RNA (lincRNA).

**Table 6:** List of differentially expressed non-coding RNAs.

Gene		
*linc-ZBTB37*	*linc-LY6H*	*linc-IGF1*
*linc-ELTD1-3*	*linc-PRSS3-4*	*linc-KCNK13*
*linc-FCGR1B-7*	*linc-HRCT1*	*linc-KIAA0513*
*linc-RC3H1-3*	*linc-VCX3B-2*	*linc-NOB1*
*linc-TET3*	*linc-GPRASP1*	*linc-ZNF583-1*
*linc-CHD1-3*	*linc-GPKOW-1*	*linc-MZF1-2*
*linc-LRGUK-2*	*linc-WNT8B*	*linc-FBXO17*
*linc-VIPR2-4*	*linc-BCL9L*	*linc-POTED-8*
*linc-AZIN1-1*	*linc-ZNF10*	
*linc-MICAL3-2*	*linc-HSCB-11*	

Additionally, 16 ncRNA genes did not appear to have any detectable fold change and were thus interpreted as completely unexpressed in the cohort of men unable to conceive. In relation to their function, most of these (11/16, 68.8%) non-protein-coding RNA appeared to guide chemical modification of other RNAs, influence methylation, and modulate stability and translation of messenger RNA. Interestingly, 13/16 (81.3%) ncRNA were located on autosomes, while only three genes were located on the sex chromosomes, following a similar distribution of spermatogenesis related genes.

## Discussion

In this study, we underline the need to perform a thorough assessment of the male gamete by sequencing the genome and epigenome to gain insight into its ability to support embryo development. While we are accustomed to determining the level of male infertility by assessing the seminal parameters in a man’s ejaculate, a semen analysis does not elucidate the ability of a man to conceive. As detailed in this work, more information can be gained by assessing the ploidy of the spermatozoon and identifying the presence of gene duplications and deletions.

It is generally believed that aneuploidy is typical of human oocyte meiosis, however, in this study we evidenced that it is also quite frequent in the male gamete as it has been known for some time [29], although the relation of sperm aneuploidy to advancing paternal age has remained a topic of debate [30]. Indeed, in men with recurrent pregnancy loss, defects of specific chromosomes are directly related to male age, as seen in previous work [29]. There are however, autosomal aneuploidies prevalently occurring in younger men [31], as in our experience for chromosome 21, where it appeared to be more frequent in 30-year-old men. This may indicate a paternal contribution to offspring with Down syndrome in relatively younger women. In our data, we also found that gonosomal aneuploidy is significantly higher in infertile patients, as previously reported [32]. Interestingly, aneuploidy is not limited to the sex chromosomes but is quite obvious also in the autosomes as confirmed by the presence of several autosomal genes involved in gamete development. Modern molecular genetic techniques carried out in couples treated by ART evidenced that those who failed to conceive had higher aneuploidy for autosomal and sex chromosomes when compared to men who successfully conceived (*P*<0.0001).

The total number of cells assessed by FISH has been chosen in order to be just sufficient to provide reliable information on the karyotypic distribution of the specimen as well as to permit the study of normozoospermic, oligozoospermic, and surgically retrieved specimens. We have chosen a threshold of 1.6% based between 2 standard deviations on donor specimens and the value obtained in patients with normal semen parameters [33, 34].

Moreover, the ability of sequencing techniques to detect CNVs provides invaluable information on the genetic profile of the entire genotype of fully mature male germ cells. CNV assessment allowed the quantification of the duplications and deletions of specific gene sequences that proved to be more appreciable in the infertile cohort of men (*P*<0.05). This allowed us to identify key gene mutations involved in the different steps of testicular function, mostly localized on autosomes. The type and cluster of genes affected in a particular group of men may help to guide toward the utilization of the most effective assisted reproduction technique to be used to address their infertility.

Male age appears to be associated with a higher occurrence of meiotic errors and chromatin fragmentation [35]. However, aging may just represent a longer exposure to a particular environmental insult and this can be best evaluated through epigenetic analysis of gene expression [36]. Among men where we sequenced sperm RNA, the group unable to conceive had a compromised expression of genes involved in nuclear compaction and acrosomal development, centrosomal function, mitochondria distribution at the midpiece, and a variance of key genes involved in the development and function of the flagellum. Finally, gene products with distinct localization within the sperm compartments informed over sperm motion and ability to engage in egg fusion, activation, and fertilization.

In this limited sample size, the analysis of RNA in spermatozoa of men with compromised reproductive outcome evidenced severely under-expressed genes involved in apoptosis and DNA repair mechanisms. This is confirmed by previous work that also identified a down-regulation of anti-apoptotic genes as well as DNA repair genes and histone modifications [37]. Their function appeared to decay with advancing paternal age and with consequent increase in sperm chromatin fragmentation, supporting the role of epigenetic screening of the male gamete. Moreover, the impaired expression of genes involved in spermatogenesis and the development of specific sperm components is capable of affecting the ability of these men to support a successful pregnancy, as supported by other investigators [38].

The ncRNAs contributed by the spermatozoon at the time of fertilization are chiefly regulatory molecules that can affect embryo development. This appears to be in agreement with previous findings that evidenced the role of spermatozoal transcripts in post-fertilization development designating them as biomarkers of male infertility [39]. Therefore, screening men for an epigenetic imbalance of ncRNA may provide crucial information on the etiology of unexplained infertility and overall reproductive capacity.

As a final effort, it remains to query the function of genes involved in immediate post-fertilization steps just prior to the activation of the embryonic genome to unravel the whole contribution of the male gamete to the development of the new conceptus [40].

Collectively, the combination of genetic and epigenetic testing provides information on the true ability of a man to reproduce when a semen analysis fails to evidence even a partial impairment of sperm parameters.

## Supporting information

S1 Table Differential expression and related p-values for genes. (XLSX)

S1 Fig Workflow of patients included in the study. (TIF)

S2 Fig Distribution plot of insert sizes from RNA-seq library prep. (TIF)
