# Supplementary figures and images for: Genetic and epigenetic profiling of the infertile male
Source: PLoS One. 2019 Mar 21;14(3):e0214275. doi: 10.1371/journal.pone.0214275 (PMC6428317; doi:10.1371/journal.pone.0214275)

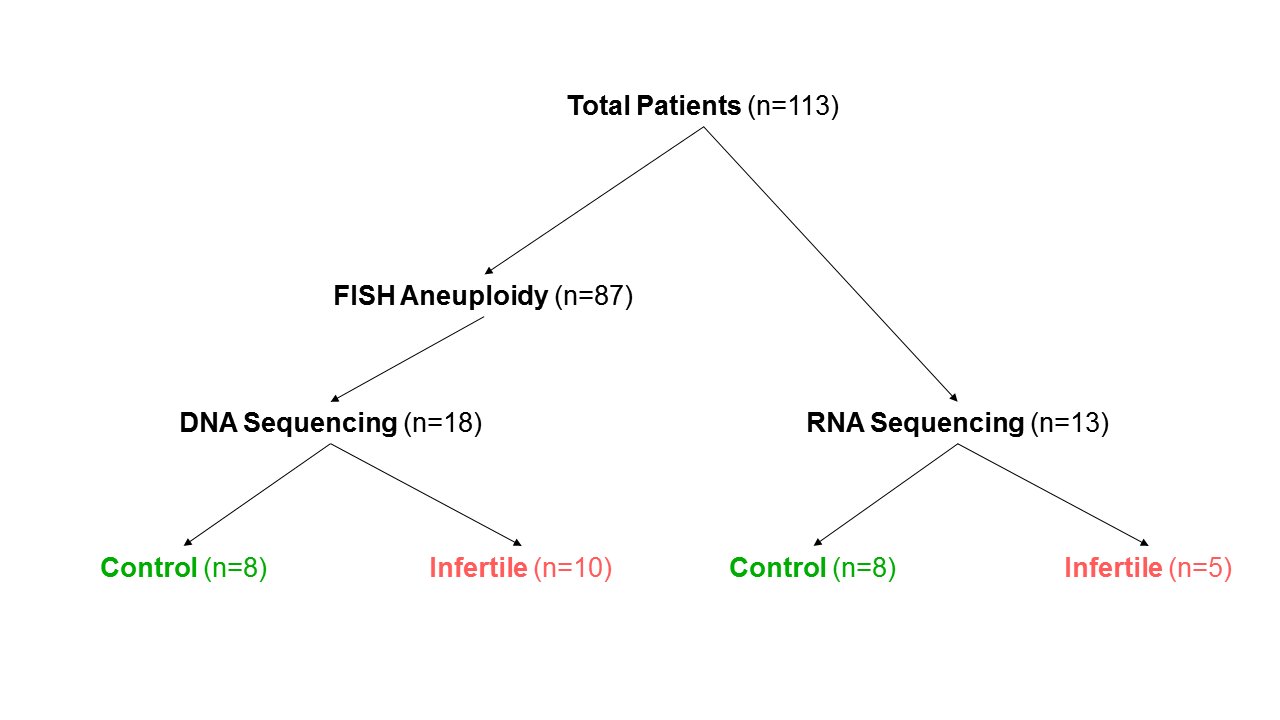

Supplement: S1 Fig — (TIF) [file pone.0214275.s002.tif]

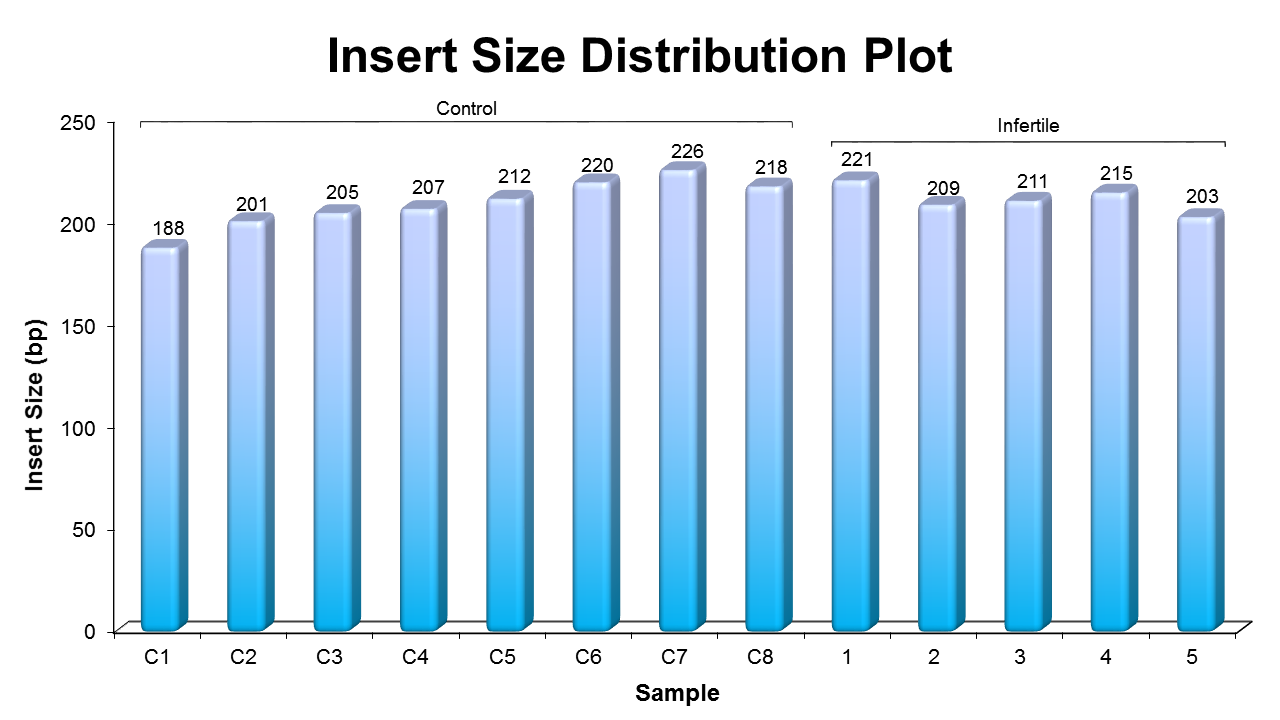

Supplement: S2 Fig — (TIF) [file pone.0214275.s003.tif]
